# Navigating HIV-Related Stigma in Switzerland: A Qualitative Study

**DOI:** 10.3389/ijph.2024.1606333

**Published:** 2024-04-26

**Authors:** Ingrid Gilles, David Jackson-Perry, Clara Le Saux, Chiara Storari, Ellen Cart-Richter, Oriana Keserue Pittet, Katharine E. A. Darling

**Affiliations:** ^1^ Centre Hospitalier Universitaire Vaudois (CHUV), Lausanne, Switzerland; ^2^ University Center of General Medicine and Public Health, Lausanne, Vaud, Switzerland; ^3^ Department of Epidemiology and Health Systems, Center for Primary Care and Public Health (Unisanté), Lausanne, Switzerland

**Keywords:** HIV-related stigma, disclosure, knowledge, qualitative methods, Switzerland

## Abstract

**Objectives:** This study sought to understand how people living with HIV experience, perceive, and navigate stigma in their everyday life and in care settings in an urban French-speaking area in Switzerland.

**Methods:** Semi-structured interviews were carried out with 19 people living with HIV in Lausanne concerning their experience of HIV-related stigma in both everyday life and in healthcare settings. Content analysis was performed to identify main and sub-themes.

**Results:** “Living with HIV” posed little or no difficulty for participants. However, the burden of anticipated and internalized HIV-related stigma played a disproportionately large role in their lives. Participants considered the general population’s low level of knowledge about HIV as problematic in this regard. While participants reported few examples of enacted stigma generally, healthcare environments were sometimes experienced as sites of prejudice and discrimination. However, some healthcare professionals were also sources of information and knowledge, contributing to participants’ “journeys of self-acceptance.”

**Conclusion:** Even in an urban environment in a country with ready access to healthcare and education, HIV-related stigma remains a concern for people living with HIV.

## Introduction

Thanks to considerable medical progress including anti-retroviral therapy (ART), people living with HIV (PLWH) can live long and healthy lives and cannot sexually transmit the virus when on effective treatment. With access to healthcare, therefore, HIV is now considered a manageable chronic condition [1]. However, HIV remains amongst the most stigmatized medical conditions in the world [2], and combating HIV-related stigma is considered central to global efforts to end the epidemic [3].

Initially theorized by Goffman as a quality or “mark” attributed to individuals that discredits them [4], stigma has long been associated with emergent, little understood, or untreatable medical conditions [5]. Latterly, conceptualizations and measures of HIV-related stigma mechanisms describe a complex phenomenon, rooted in both societal perceptions and behaviors, and the perceptions of PLWH [6]. Earnshaw and Chaudoir, for example, class stigma mechanisms for PLWH as: “enacted” stigma (experiencing discriminatory behavior, stereotyping, and/or prejudice from others due to HIV status); “Internalized” (endorsing and applying to oneself negative stereotypes associated with HIV), and “anticipated” stigma (expected HIV-related stereotyping, and/or prejudice from others) [6‐8].

These mechanisms, and resulting discrimination (prejudicial individual, legal, or institutional treatment), lead to negative outcomes for PLWH, impacting mental, social and physical health [6, 8]. HIV-related stigma negatively impacts adherence to ART [9] and impedes access to healthcare provision and HIV services [10, 11]. Anticipated HIV-related stigma and negative stereotypes about PLWH amongst HIV negative populations are linked to low testing [12], contributing to late diagnoses of HIV and undermining early treatment initiation [13]. HIV-related stigma therefore threaten the health and wellbeing of PLWH, and the entire cascade of HIV prevention and care.

A systematic review of qualitative literature (*n* = 27) concerning HIV-related stigma [14] found anticipated stigma to be a barrier to disclosing HIV status. However, when participants did discuss their status with their families or close social circles, the outcome was often positive, bringing a sense of liberation and acceptance. Internalized stigma manifested through unnecessary fear of transmitting HIV to participants’ social and intimate circles. Creating networks of other PLWH was proposed as a mitigating strategy.

A synthesis of qualitative research (*n* = 55) specifically considering HIV-related stigma and health [5] noted a range of healthcare practices perceived as stigmatizing or discriminatory, including violations of confidentiality, segregated healthcare environments, “judgementalism,” and excessive precautions taken by healthcare professionals (HCP). Strategies for navigating stigma included developing connections with other PLWH through peer-support programs, although some avoided this as an unwelcome reminder of their HIV status. However, sharing one’s HIV status openly was often experienced as empowering, with some PLWH transforming their experiences of stigma into opportunities for empowerment and change.

In Switzerland approximately 17,000 people live with HIV [15], of whom about 1,200 are treated at Lausanne University Hospital. Few studies specifically address HIV-related stigma in a Swiss context. In one quantitative study (*n* = 5,563) (manuscript submitted), stigma was reported by 89% of participants living with HIV in Switzerland. A smaller quantitative study (*n* = 72) [16], examining the living conditions and quality of life of PLWH aged over 50 in Switzerland, found that 47.2% of respondents had experienced discriminatory practices mostly in clinical settings or workplaces, from insurance agencies, or public authorities denying them travel visas. Among PLWH enrolled in the Swiss HIV cohort study between 2009 and 2012, 49.8% were late presenters (LP: people presenting for HIV care with a CD4 count below 350 cells and/or an AIDS defining event) [17]. A study seeking to understand the reasons for this high rate of LP found “fear” to be an important contributory factor, with 39% of participants fearing their relatives’ reactions, and 26% their partners’ reaction to a potential HIV diagnosis [17].

Whilst previous qualitative studies examining HIV-related stigma in Switzerland exist [18], there is no recently published qualitative research exploring the HIV-related stigma among PLWH in Switzerland in the era of modern ART and the U=U campaign (“Undetectable” = “Untransmissible). The objective of the current study was therefore to understand how PLWH experience, perceive and navigate HIV-related stigma in Switzerland today including in care settings; to qualify the burden this might represent, and to understand the strategies put in place to navigate it.

## Methods

### Design

We conducted semi-structured interviews between March and May 2022 with 19 patients from the Lausanne University Hospital Infectious Diseases Outpatient Service (LUHIDOS). The interviews lasted between 60 and 90 min, could be interrupted at any time by participants, and were conducted at the psychosocial wing of the service, largely used for peer mentoring, therapeutic patient education, and other support services by and for PLWH. Virtual interviews were also possible, and two participants chose this option. The study was conducted in accordance with the criteria for good practice in qualitative research (see. The COREQ checklist in Supplementary Material S1).

### Patient and Public Involvement (PPI)

Research indicates the importance of community involvement in research processes, and the reporting of that involvement [19]. A member of the service’s council of PLWH was part of the research team throughout the research process, notably concerning design of information and interview tools, interviewers’ training, data analysis, and presentation of results.

### Recruitment

PLWH treated at LUHIDOS were invited to take part in the study by a senior clinic nurse when attending for routine blood tests, or by their physician. Both clinic nurses and physicians had access to the medical notes of potential participants and approached only patients who met the study eligibility criteria. Patients were eligible if they were older than 18 years, had an adequate level of French and were enrolled in the Swiss HIV Cohort Study network, a representative systematic longitudinal study enrolling PLWH treated in Swiss University Hospital infectious diseases outpatient services, large cantonal hospitals, and SHCS affiliated private clinicians [20] Patients with severe depression or neurological impairments (according to their medical records) were not approached for participation by the clinic nurse or their physician. All patients who were eligible and interested in the study were contacted by the study physician (KEAD) in person or by telephone. This initial contact served to confirm study eligibility, notably, the absence of psychiatric comorbidity or neurocognitive impairment, to explain the study and answer any questions. Eligible patients then received an information letter with a written consent form (template from the local ethic committee) including consent for the use of their medical records, their participation in the study, and the recording of interviews. A period of at least 72 h was required between receiving the information letter and signing the consent form. The study physician in charge of recruitment was not involved in interviews and had no access to interview transcripts. The three researchers who conducted the interviews did not work at the consultation and had no access to patients’ medical records or identifying information. Judgment sampling (a non-probabilistic sampling method used in qualitative research, in which researchers use their knowledge and judgement in selecting sample members [21]) was applied to achieve maximum participant heterogeneity particularly regarding ethnicity, time since diagnosis, education level, gender and mode of transmission. Recruitment stopped when data saturation was reached. Participants were remunerated for their time and travel expenses.

### Data Collection

An interview guide (see Supplementary Material S2) was constructed from existing literature on HIV stigma and through discussion within the research team. This guide, although taking up elements of Earnshaw and Chaudoir’ model [8] on HIV-related stigma, proposed broader probes, in particular concerning social representations of HIV and strategies put in place by participants to deal with stigma. This allowed us to adopt an inductive rather than a deductive method of collecting information, an approach we felt to be important given the paucity of data on this issue in Switzerland. Interviews were conducted in French, by three external female researchers trained in qualitative interview techniques (CLS, CS, and OKP). Three sessions were organised before the interviews to train the interviewers to use appropriate terms, to acquire basic knowledge about HIV, and to be familiar with technical terms that participants might use during interviews. Sessions were led by a member of the service’s council of PLWH and a professional from the psychosocial unit of the LUHIDOS, who also lives with HIV. Given the sensitivity of the topic, a specialist nurse was on hand during or following interviews to support participants in case they experienced emotional distress. Interviews were audio-recorded and fully transcribed in French.

### Interviews Analysis

Interview transcripts were analysed using the IRaMuTeQ software (version 0.7 alpha 2, 2008–2014 Pierre Ratinaud), a computer assisted qualitative data analysis software. This type of software, specifically intended to conduct inductive qualitative-content analysis [22], including in the domain of health perceptions and HIV [23‐25], allows identification of recurring themes using word or expression co-occurrences. It extracts underlying common narrative structures from a body of textual data, following the Reinert method [26], whereby the software partitions each interview into elementary contextual units (ECU; i.e., sentences) which serve as units for the analysis. Two hierarchical descending classification of words by ECU are carried out on all the interviews to generate a classification of words in thematic classes and a tree graph showing associations between these classes in the texts. Each extracted thematic class is associated with a typical vocabulary, and typical extracts to facilitate the identification and labelling of themes. Once the software classification is completed, the researchers identify and label the classes according to the typical words and extracts. Two researchers specialized in qualitative methods and in the use of this software were involved in interviews analyses. Researchers first conducted their analysis separately, and then jointly reached a consensus on the labelling and interpretation of thematic classes. Two half-day sessions were organized with the researchers involved in the interviews and the member of the service’s council of PLWH, all having read the anonymized interviews, to ensure that the analysis covered all the issues raised in the interviews, to interpret thematic classes and to reintroduce verbatims into the analysis.

Time since diagnosis, gender, ethnicity and education were considered in analysis.

Ethics approval was obtained from the Local Ethics Committee (Project-ID: 2021-02224).

## Results

### Participants

A total of 40 patients treated at the LUHIDOS were approached to participate in the study. Eleven agreed but did not meet the inclusion criteria or did not attend the information meeting with the study physician, and 10 decided not to participate after the information meeting with the study physician. Nineteen people living with HIV participated in the current study (Table 1). All were receiving antiretroviral therapy, 11 were women, 15 were Swiss or from European Union countries; four were non-European (Africa, South America), 13 had stopped studies before high school, 16 had been diagnosed for more than 10 years and 10 had CD4 nadir score <200 cell/mL. Mean age was 49 years.

**TABLE 1:** Characteristics of participants. (Navigating HIV-related stigma in Switzerland: a qualitative study. Switzerland 2022).

Characteristics	*n* (%)
Gender	
Female	11 (57.9)
Male	8 (42.1)
Age (years)	
18–29	1 (5.2)
30–39	4 (21.1)
40–49	2 (10.5)
50–59	8 (42.1)
60–69	4 (21.1)
Nationality	
Swiss	12 (63.2)
Non-Swiss European	3 (15.8)
Non-Swiss, Non-European	4 (21.0)
Education (completed)	
Up to mandatory (around 12 years)	2 (10.5)
Apprenticeship/technical school	11 (57.9)
High school	4 (21.1)
Superior education (e.g., University)	2 (10.5)
Time since diagnosis	
More than 10 years	16 (84.2)
10 years and less	3 (15.8)
CD4_nadir cell/mL (mean)	233 (min = 11; max = 704; SD = 177.5)

### Thematic Content Analysis

**FIGURE 1:**
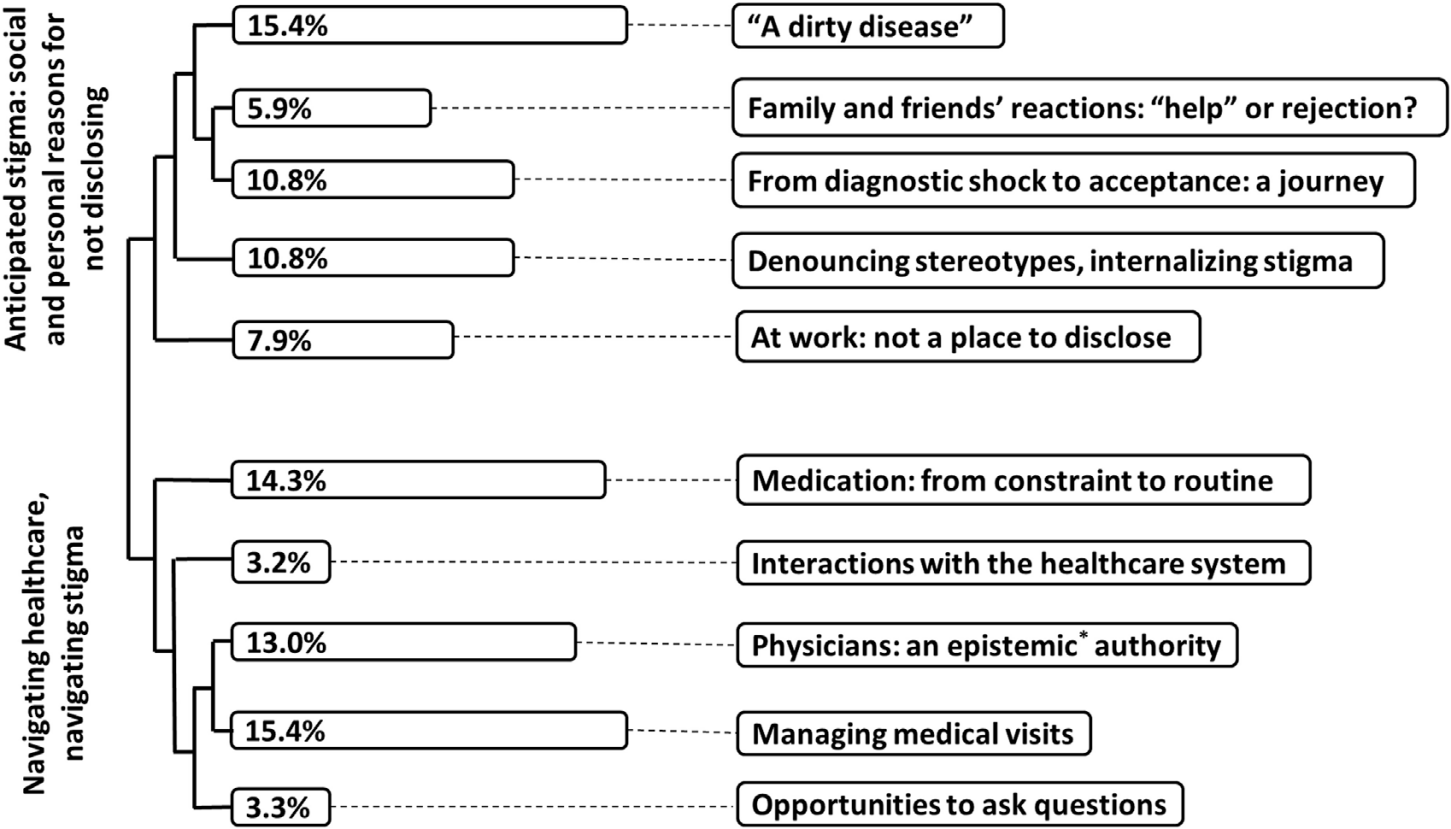
Themes, subthemes, typical excerpts from the content analysis. Percentages correspond to the proportion of text included in each subtheme. *Epistemic: relating to knowledge or to the degree of its validation. (Navigating HIV-related stigma in Switzerland: a qualitative study. Switzerland 2022).

HIV-related stigma was evoked by participants through two main themes. The first main theme concerned anticipated stigma and the social and personal reasons why participants were reluctant to talk about their HIV status. The second main theme was about *Navigating healthcare, navigating stigma*, and concerned the experience of stigma in healthcare settings through interactions with healthcare professionals. While the first main theme was overrepresented among men, participants from Switzerland or European countries and those having a higher level of education, the second was mainly evoked by women, participants from non-European countries and participants with a lower level of education. These two main themes and related sub-themes are described below and appear in Figure 1.

#### First Main Theme: Anticipated Stigma: Social and Personal Reasons for not Disclosing

This main theme was composed of five sub-themes (in italics in the text). These five sub-themes illustrated how anticipated stigma had such a powerful impact in participants’ lives, determining whether or not they disclosed that they live with HIV. Most participants sought to avoid potential stigma by not disclosing their HIV status. Although few participants noted experiences of enacted stigma in their social or intimate lives, this non-disclosure constituted an important part of their lives, and all had strategies to decide who to disclose. In the following section we provide details of the five sub-themes. Illustrative quotes are presented in Table 2.

**TABLE 2:** Example of quotes illustrating sub-themes from the anticipated stigma: social and personal reasons for not disclosing main theme. (Navigating HIV-related stigma in Switzerland: a qualitative study. Switzerland 2022).

Sub-themes	Illustrative quote
« A dirty disease »	“People think that it is a disease of, sorry for the terms but of a whore, of someone dirty, in fact we could say the dark side of people.” (Woman, 26 years old, born with HIV)
	“People … are still … afraid of getting sick, afraid of catching it and are not convinced, no matter how much we tell them, that it’s only transmitted that way, they’re never one hundred percent sure, even if we tell them” (Woman, 59 years old, diagnosed > 10 years)
Denouncing stereotypes, internalizing stigma	“I think that mentalities have not evolved but in my opinion, it is because it was linked to sex and drugs in the beginning … one question I ask myself is how we could make people realize that now HIV has evolved a lot” (Man, 31 years old, diagnosed< 10 years)
	“And then it’s how you actually get it, by having unprotected sex, by cheating, or by taking drugs, and I clearly didn't belong to any of these categories[…] So it’s still a category that is a bit marginal, well not really part of normal society. The drugs, well, things like that, whereas …at that time perhaps it was that, but there were still a few [people living with HIV] for whom it wasn't that at all, but those people, well, perhaps they hid it, we didn't talk about it so much and as I say, in the waiting room, every time I was all alone…I would come in and I would say, well, damn, I am all alone.” (Woman, 51 years old, diagnosed > 10 years)
	“Yes, for me it’s a matter of trust, after, most of the people I told either forget because it’s just another piece of information and that suits me, and on the other hand, for those I told, yeah, for all those I told, they reacted very well, I had a sort of heart attack every time I told them and I started to cry because I was afraid of losing that friendship, but in fact no, it always went well […] My in-laws don't know and it’s going to stay that way. Because they may be open-minded, but for some things I think they’re really closed-minded, so I don't want to tell them because they are part of my life and I’m part of theirs.” (Woman, 26 years old, born with HIV)
Family and friends’ reactions help or rejection	“Maybe your family will help you… the people who are very close to you will help you to get over it, but we don't know how they will see you afterwards. Will the way they see you change or not? We don’t know in fact, we don’t know, that’s what it is.” (Woman, 35 years old, diagnosed for 10 years)
From diagnostic shock to acceptance: a journey	“A lot of people were trying to fight HIV in their heads, I mean a bit more psychologically, but I decided to live with it … I said to myself: anyway I can’t do anything, I can’t fight it but I’m going to live with it…” (Man, 66 years old, diagnosed > 10 years)
	“Testimonies are a way to educate future generations.” (Man, 66 years old, diagnosed > 10 years)
	“But I find that we tend to show people who are very thin, so yes, we can have problems of lipodystrophy […] I was a bit out of that category, but maybe people like me are not shown because they don’t want to show themselves.” (Woman, 51 years old, diagnosed > 10 years)
At work: not a place to disclose	“My boss mustn’t know anything about it, especially as I don’t see what difference it makes to my work, I work quite normally […] I have a lot of colleagues who haven’t finished school … and I think they wouldn’t understand.” (Man, 56 years old, diagnosed > 10 years)

The two first sub-themes concerned broad social perceptions of HIV with the word “people” being overrepresented. First, participants evoked the fact that HIV is still perceived as “*a dirty disease*” by “people” (a generalized word used during interviews to describe social attitudes) as it was in the 1980 s. They emphasized two aspects: their awareness of the demeaning nature of social perceptions of HIV and the fact that this has not evolved despite changes in the medical and social realities of HIV. Participants noted that “people’s” poor knowledge of HIV today was a barrier to them being able to change their perceptions. Participants therefore expected to be rejected if they revealed they lived with HIV. Assessing the HIV-related knowledge of the people they were dealing with, or their ability to assimilate this knowledge, was thus described as one of their main strategies for deciding whether or not to disclose their HIV status: the higher the level of recent HIV-related knowledge, the less likely the PLWH felt they were to face stigmatizing or discriminatory behavior.

The second sub-theme, was labeled “*Denouncing stereotypes, internalizing stigma.*” In this sub-theme, participants evoked stereotypes from the “80 s (“homos,” “junkies,” “whores”) and noted that they still applied to PLWH today. Once again, participants explained this by the ignorance of “people” about advances in HIV treatment and the new realities of living with HIV.

In this sub-theme we noted tensions in participants’ discourse, reflecting mechanisms of internalized stigma. The first tension concerned participants’ self-identification with the group of PLWH. Participants all agreed that life with HIV had evolved towards a kind of normalization, with some even mentioning the fact that HIV was now less burdensome than other chronic illnesses such as diabetes. However, a majority (mainly women and heterosexual men) were reluctant to define themselves as part of the group of “people living with HIV,” fearing association with the above prototypes and the anticipated resulting social rejection. The second tension apparent in participants’ discourse was between their knowledge and their attitudes towards intimacy: despite being on effective treatment, and knowing that they could not transmit the virus to sexual partners, participants still feared such transmission, with some still using condoms as a result. In a third sub-theme, *Family and friends’ reactions: “help” or rejection?,* Participants expressed their “need” to talk about HIV to those close to them. Being accepted as living with HIV by family and friends was part of personal acceptance for some participants, a way to “get through it.” However, family ties or friendships alone were not considered to guarantee acceptance, with the fear of rejection always present when they disclosed their HIV status. The main strategy for deciding when (or if) to disclose to family and friends about their diagnosis was not HIV-related knowledge but open-mindedness and, particularly for friends, trustworthiness. Knowing that friends would not disseminate the information was paramount to participants.

The fourth sub-theme, *From diagnostic shock to acceptance: a journey* concerned participants’ personal acceptance of living with HIV. They described it as a “journey” bringing to the detachment from stigma and from giving importance to other people’s gaze. Here again, knowledge had a special status: accepting life with HIV meant distinguishing between what was medically related to HIV and what was socially related to HIV. Here there were two opposing discourses. For some, HIV was essentially a question of stigma. This discourse generally came from people at the beginning of their “journey,” to whom not to disclose, and keeping track of “who knows and who doesn’t know” were the principal constraints linked to living with HIV. For others, stigma was a secondary aspect of HIV. In general, the latter had learnt to accept living with HIV and had told more people their HIV status. Accepting living with HIV also implied teaching and sharing their knowledge with others. The public testimony of people living with HIV was described as an important factor in changing mentalities. For participants, HIV was no sufficiently mediatized and when it was, testimonies came from members of militant groups, generally gay men, which, in their opinion, risked maintaining some stereotypes about people living with HIV. Participants regretted that there was not a wider variety of people testifying, although many said that they themselves would not testify, for fear of the implications of that visibility.

The fifth sub-theme was labeled “*At work: not a place to disclose.”* The workplace was described as the place where participants most anticipated stigma and did not intend to discuss HIV. Most participants’ colleagues were not aware that they lived with HIV. Their main fears were that colleagues would see them differently, “talk badly” about them, or perceive them as less competent and effective at work. In one case, a participant explained that she had met a colleague at the HIV service and realized that they were both living with HIV, but at work they continued to act as if nothing had happened; this common experience did not bring them closer. Paradoxically, participants who disclosed their HIV status at work reported generally positive reactions and considered that disclosure had strengthened relationships with colleagues. As with family members, open-mindedness and trustworthiness were important criteria to decide whether to tell colleagues or not.

#### Second Main Theme: Navigating Healthcare, Navigating Stigma

This second main theme was composed of five sub-themes (see Table 3 for illustrative quotes), which globally gathered descriptive discourses about their healthcare-related experiences generally and as it related to enacted or anticipated stigma.

**TABLE 3:** Example of quotes illustrating sub-themes from the navigating healthcare, navigating stigma main theme. (Navigating HIV-related stigma in Switzerland: a qualitative study. Switzerland 2022).

Sub-themes	Illustrative quote
Medication: from constraint to routine	“I will always need these medicines […] I take them in the evening, I try not to forget. But then it really goes like clockwork, it’s easy, I take them, it’s become kind of automatic, when I brush my teeth I take them and that’s it.” (Women, 59 years old, diagnosed > 10 years)
	“I take four pills a day, which doesn't interfere with my life. I take my pills like someone who has other illnesses, high blood pressure or whatever. But I find that we tend to show skinny people, so yes, you can have problems with lipodystrophy, but I’ve often hidden it, I’ve had an operation but that’s it, I don't feel like…” (Women, 51 years old, diagnosed > 10 years)
	“I bring my empty boxes back here to the hospital pharmacy, because I say to myself: that, in the garbage, if suddenly the cat in the neighborhood pulls it out, it’s not possible.” (Woman, 50 years old, diagnosed > 10 years)
Managing medical visits	“For the moment it has always gone well. When I go to give blood, I like to talk to the nurses, they are always ready to listen, we talk about everything, about life, about what’s going on.” (Woman, 52 years old, diagnosed > 10 years)
	“I go to these appointments; I try to get them done as quickly as possible because I don’t like going there I don’t like going there because it reminds me too much of this illness so I tend to cut it short.” (Woman, 59 years old, diagnosed > 10 years)
Opportunities to ask questions	“I think that now we should also make sure that the insurance companies are not discriminating. […] I also think that insurance companies should no longer be able to refuse someone for complementary insurance.” (Man, 59 years old, diagnosed > 10 years)
	“If I have any doubts, I know I can ask [the doctor] and I’ll get the right answer. Even with the nurses, I know I can ask them questions, and if they don’t know, they’ll ask the doctors, and otherwise I have a really good relationship.” (Woman, 43 years old, diagnosed > 10 years)
Interactions with the healthcare system	“But that’s pretty cruel too, I find, in the Swiss system, to see that not only are you sick, but you have to pay for it, I find that pretty difficult. For me, it was difficult at first when I arrived in Switzerland to understand… The fact that, in the end, you have to take a drug that costs a lot, so you have to pay for it, you have to take maximum coverage with a minimum deductible, so you have to pay for the whole thing in the end … ” (Man, 66 years old, diagnosed > 10 years)
	“I remember an insurance representative came to the house and wanted to know if we wanted to take out a complementary insurance, but I know I’m not allowed to with … so he wanted us to fill in a form and I said I wasn’t willing to fill it in. It’s annoying, it’s a shame that nothing is being done about it, nothing is changing. Even if you know that your illness has been stabilized by medication, I mean, you’re only entitled to basic insurance and that’s really all you get.” (Woman, 43 years old, diagnosed > 10 years)
Physicians: an epistemic authority	“I trust doctors … If they tell me to do it this way I won’t do it any other way”. (Woman, 51 years old, diagnosed > 10 years)
	“As for the doctors, I have to talk openly… I have to, I am the patient. […] In the past it wasn’t easy … It was very, very difficult… I didn’t accept it. […] It was a surprise and it’s from talking to doctors […] with experienced doctors, I listen, they explain, it’s a lot, you know, it reassures you.” (Woman, 61 years old, diagnosed > 10 years)

In a first sub-theme—*medication: from constraint to routine*—participants described frequencies and moments in the day at which they took their medication. What was important here was the repetitive aspects of taking medication, and the automatism or “daily routine” that characterized it. Medication was seen on the one hand as a vehicle for stigmatization, whereby having to take a medication or having a box of pills visible represented a significant risk of involuntary disclosure: medication was then perceived as a constraint. On the other hand, taking medication was a way to keep away visible signs of HIV, equated with good health and not looking sick. Consequently, medication here was perceived as less constraining. Regardless, participants had developed strategies to hide medication, such as keeping it in packaging of more “value-neutral” conditions. The four remaining sub-themes related to medical visits or to participants’ interactions with the healthcare systems.

Two other sub-themes, *managing medical visits* and *opportunities to ask questions*, were purely descriptive, concerned with frequency of visits to the hospital, concrete interactions with healthcare professionals and the fact that the hospital was a place they could ask questions and collect information about HIV or other health-related issues. Here, stigma was only indirectly evoked. Participants explained that most healthcare professionals with whom they had contact knew about their HIV status. Generally, participants did not question whether to disclose or not in care settings feeling it was necessary. However, some did report discriminatory behaviors from healthcare professionals such as appointments fixed only at the end of the day or unnecessary protective measures. More frequently, participants reported inappropriate questions or remarks about how they had come into contact with HIV, which they considered to be irrelevant to the consultations. When participants did report stigmatizing or discriminatory situations or prejudice in healthcare settings, they tended to attenuate their seriousness by finding justifications for professionals’ reactions (e.g., “it’s more convenient for them,” “it wasn’t a good day,” “maybe he needed to know”).

In a fourth sub-themes participants evoked *their interactions with the health system* more globally. They often perceived the system as overly complex and disadvantageous for people living with HIV. Communication of personal information was a sensitive aspect, with participants expressing doubts as to when the HCP needed to know for medical reasons versus when they were simply curious.

The last sub-theme—*physicians: an epistemic authority* - specifically concerned the relationship with physicians and particularly HIV physicians. This relationship was described as respectful and trustworthy, and indeed respect and trust were the prerequisites for care adhesion. However, for some participants this relationship was also marked by deference to their physicians and a passivity towards decision taking.

In this regard, physicians were perceived as holding considerable epistemic authority, being perceived as a reference for information or decisions related to HIV at the medical level, but also at the personal level and on how to manage HIV-related information.

Several participants explained that their decision not to disclose their HIV status came from discussions with their physician (“my physician told me not to talk about it”), usually at the time of diagnosis or during initial consultations. Moreover, the frequent changes of doctors at the hospital did not provide an opportunity to discuss stigma or disclosure on an ongoing basis. To disclose or not their HIV status more widely was thus set at the point of diagnosis as a rule to follow, and rarely discussed later.

## Discussion

In our study, participants reported little or no difficulty living with the medical side of HIV whereas living with HIV-related *stigma*—and more particularly although not uniquely, anticipated stigma was the real burden. This echoes the importance given by international bodies and the literature more widely to fighting stigma, [27]. Our findings clearly indicate that even in Switzerland, a country with ready access to treatment and a high level of education generally, HIV-related stigma casts a pall over the lives of PLWH. This is, however, neither immutable nor a fatality, and participants described living with HIV in terms of a journey to self-acceptance. People who are “further down the road” of this journey have learnt who to tell about their status and are comfortable with this, no longer expressing the fear that someone will find out about their HIV status without their consent. Confirming results elsewhere, self-acceptance concerning living with HIV appears to reduce the weight of anticipation for participants here [28].

Acquiring knowledge and experiences about HIV and about living well with HIV was perceived by participants as an important part of this process: unsurprisingly, then, information has a special status for people living with HIV, and it is necessary for them to understand HIV in order to accept living with it. The most common way to achieve this is to talk with their physician. Indeed, while healthcare professionals and more particularly physicians are seen as possessing medical knowledge, participants’ confidence in their physicians goes further, and they are also trusted regarding navigating stigma [29]. Decisions about disclosure are sometimes taken together and physicians may seek to protect their patients by advising them not to disclose their status. There is a potential paradox here: this “protective silence” [30] could reinforce the idea that HIV is taboo and should not be discussed. Indeed, contradicting the notion that keeping HIV secret is protective, while revealing one’s HIV status carries some risks, it is also associated with reduced anxiety and increased access to knowledge and social support, all important steps in reducing anticipated stigma [31].

Other routes to knowledge acquisition exist, such as peer discussion groups or mentoring programs. Participants in our study recall being offered opportunities to develop contacts with other people living with HIV which some refused, appearing unwilling to be associated with other people who may embody negative stereotypes. Participants’ resistance to being associated with other stigmatized people is a barrier to discussing their HIV status which has been noted elsewhere [30]. Through internalized stigma, participants may deny themselves the very social support which has been shown to be a protective factor against stigma [32]. This finding echoes research showing that people who are able to conceal a stigmatized identity are less likely to frequent ‘similar others’ than people with more visible stigmata, thereby benefitting less from the potential positive effects of group membership.

Importantly, throughout interviews, relatively few participants here reported experiences of enacted stigma or negative reactions when they revealed their status. Despite this, anticipation and avoidance played a disproportionately large role in their daily lives. Fear of discrimination, shame and associative stigma—the most commonly reported reasons for not disclosing—were so pervasive that even participants’ positive experiences were not enough to mitigate that fear, and strategies to cope with the necessity of informing others of their status were paramount in their life’s organization, involving complex systems of control. These findings are supported by other research in the field suggesting that people living with HIV may report more experiences with anticipated than enacted stigma [33]. Anticipated stigma can have important negative impacts on psychological wellbeing [34], and is more highly associated than enacted stigma with, for example, low treatment adherence and physical symptoms of HIV [33, 35, 36]. Findings here of low reporting of enacted stigma and high reporting of anticipated stigma do however contradict some existing research suggesting that the former is a principal source of the latter [37].

Our finding suggest that, although enabling people living with HIV to move towards acceptance is beneficial for them, interventions solely targeting PLWH would a) delegate the burden of reducing stigma to PLWH and b) insufficient to tackle the issue of the HIV-related stigma, given the importance of knowledge on the part of all social actors in this respect. Joint actions should therefore be taken at different levels beyond the individual. The Health Stigma and discrimination framework proposed by Stangl and others’ [10] advocates for such “multi-component interventions”. According to this framework, stigma cannot be reduced to a dichotomy between people who experience stigma and people who perpetrate. The consequences of stigmatisation affect not only people who are stigmatised, but also the population as a whole and the way organisations and institutions function. Participants regretted, for example, the lack of large communication campaigns, including a diversity of PLWH, to educate people about HIV. To be more effective, the actions taken to address HIV-related stigma should therefore target different levels or actors. Healthcare-related stigma is a good example. In our study, experiences of enacted stigma that participants reported occurred in care settings (in general other than HIV care settings), with for example, disclosure issues, inappropriate questions about acquisition of HIV, or unnecessary protective measures. This phenomenon has been widely reported in the literature [38, 39] with weak evidence of efficacy regarding interventions at the individual or group level such as professionally-assisted peer group interventions or workshops comprising didactic lectures [39]. More efficient interventions would consider stigmatization as a larger process encompassing all actors and could for example, include training sessions with professionals and PLWH or introducing institutional monitoring tools that would give visibility to institutional policies aimed at stigma reduction.

### Limitations

The study exclusion criteria precluded access to people living with HIV in vulnerable situations (e.g., homeless people, people who use intravenous substances) and to patients with depression or cognitive impairment. Given that people accumulating vulnerable situations and identities might experience multi-layered stigmatization and given the established link between stigma and depression [40], it is possible that exclusion of people on this basis lends bias to results. Similarly, the fact that 16 participants out of 19 had been diagnosed with HIV more than 10 years ago could represent a bias as they may have experienced more stigma than people living with HIV for a shorter period of time [41]. However characteristics of participants largely reflected characteristics of people attending the HIV consultation in Lausanne, and so results might provide an understanding of HIV-related stigma experienced by this specific population. Moreover, qualitative study does not aim at providing an understanding of a phenomenon generalizable to different populations but rather a transferable understanding [40]. The fact that most of our results are congruent with past literature speaks in favour of this transferability.

### Conclusion

This study confirms that stigma is still a salient issue for people living with HIV, even in Switzerland. More particularly, navigating anticipated stigma constitutes a considerable burden for people living with HIV, even though few reported actual experiences of stigmatization. Anticipated stigma can be at least as damaging as enacted stigma, and so both should be considered when it comes to improving the quality of life of people living with HIV. Healthcare professionals, often seen as an all-knowing authority, sometimes advise patients not to discuss their HIV status, particularly at the time of diagnosis. Despite the good intentions behind this advice, it may be damaging rather than protective, unless it is followed up on in later consultations: some people living with HIV find that talking about their HIV status with people other than healthcare professionals can be beneficial and empowering. What was useful for a patient at the time of diagnosis—keeping their HIV status to themselves to give them time to think and adapt—may in fact become a contributory factor to exacerbating anticipated stigma at a later point in their trajectory. While meeting other people living with HIV and participating in peer-projects has been shown to be an effective way of reducing anticipated stigma, it seems that some people living with HIV may avoid this, through internalized negative stereotypes. To reduce stigma of all types, multiple interventions targeting not only PLWH but also healthcare professionals, healthcare system stakeholders (including insurance companies), politicians, and the public, are needed. HIV needs to be brought back into the public domain and discussed, particularly concerning advances in treatment and modes of transmission. Increased public visibility of a wider range of people living with HIV, through testimonies could also be central.
