# Supplementary material for: Navigating HIV-Related Stigma in Switzerland: A Qualitative Study
Source: Int J Public Health. 2024 Apr 26;69:1606333. doi: 10.3389/ijph.2024.1606333 (PMC11082645; doi:10.3389/ijph.2024.1606333)
Supplement: Supplementary file 1 [file DataSheet2.PDF]

Primer 1 : general

To begin, can you tell me what made you agree to participate in this study?

Probes: "As you may have read in the newsletter and as I reminded you earlier, our study is about HIV-related stigma. When I talk to you about HIV-related stigma, what does that mean to you, what would you say about it?

"What did you think when you were told about this study? Can you tell me why? »

Themes

- Public' perception of people living with HIV and anticipation of stigma

Can you tell me how you think people perceive HIV and people living with HIV? What issues might you encounter related to this perception? What impact might this have on your life in general (detail social/work/family if necessary)?

- Hiding the disease and concerns about talking about it (With whom, why, enabling contexts)

Who have you told that you are living with HIV? What made you tell? To whom or in what context do you not tell? What makes you not tell these people or contexts? What might happen if it were known that you are living with HIV in these contexts (name the ones mentioned)?

- Temporality (changes in anticipation or perception since diagnosis, changes in attitudes of "others," changes in attitudes of relatives and family)

How has what you think or feel about stigma changed since you have been living with HIV? if not changed: How do you explain that it has not changed over time?

- Treatment and stigma (adherence, visibility of side effects and treatment strategy, U = U...)

Can you describe to me how you manage every day with your treatment?

- Self-image - internalization (including impact on intimate relationships with partners)

How do you see yourself today? What would you say if you were asked to introduce yourself?

- Actual experience of stigma

When you think about stigma, are there specific events or examples you have experienced that come to mind? Can you describe them to me? How did you respond?

- Strategies for dealing with or avoiding stigma

What helps or has helped you deal with or avoid stigma? or Tell me how you learned to deal with it?

- Absence of stigma (who, why, what contexts support...)

Are there people around you who have remained the same after learning that you are living with HIV? Why do you think they did not react like everyone else? Can you describe the context in which this happened? How did you react?

What didn't we discuss that I should know about to better understand HIV-related stigma?

Primer 2 : healthcare

In our research we are also interested in stigma in care or in access to care. What can you tell us about that?

How would you describe how this manifests itself? Can you give me some examples? What did you say to yourself at the time?

Themes

- Specific care settings in which participants feel or experienced stigma

Could you describe a situation you have experienced with one or more health care professionals in which you felt stigmatized? What are the care settings in which you have most often had these problems? What type of professional do you tell that you are living with HIV and who do you not?

- Types of stigma (disclosure, always last)

How do you think stigma affects care? Can you think of any examples of stigmatizing situations that might occur in the hospital or with a doctor or health care professional?

- Impact on care

What consequences can this have on people living with HIV? in relation to their health?

- Suggestions for improved management

What could be done to reduce stigma in care?

What haven't we discussed that I should know about to better understand stigma in care or access to care?

Closing Question

What haven't we discussed that I should know about to better understand HIV stigma?

We are coming to the end of the interview. We have talked together about HIV-related stigma in everyday life and in care. In addition to understanding a little better what it is like to be confronted with stigma, we would like to create guidelines that would help professionals to better address this topic with their patients during consultations or so that they are careful not to induce stigma. In your opinion, what would be the concrete and essential elements to be included in these guidelines?

End of the interview

Please be aware that if you feel the need or desire, you can discuss HIV-related stigma with a nurse specialist (SN) from the clinic who is familiar with the issue. [Give contact information if interested]
